# Advantages and disadvantages of the use of the CSF Amyloid β (Aβ) 42/40 ratio in the diagnosis of Alzheimer’s Disease

**DOI:** 10.1186/s13195-019-0485-0

**Published:** 2019-04-22

**Authors:** Oskar Hansson, Sylvain Lehmann, Markus Otto, Henrik Zetterberg, Piotr Lewczuk

**Affiliations:** 1 Clinical Memory Research Unit, Department of Clinical Sciences, Lund University, Malmö, Sweden; 2 Memory Clinic, Skåne University Hospital, Malmö, Sweden; 3 Center of Excellence for Neurodegenerative disorders (COEN) of Montpellier, Montpellier University, CHU Montpellier, INSERM, Montpellier, France; 4 Department of Neurology, University of Ulm, Ulm, Germany; 5 Clinical Neurochemistry Laboratory, Department of Psychiatry and Neurochemistry, Institute of Neuroscience and Physiology, The Sahlgrenska Academy at the University of Gothenburg, Gothenburg, Sweden; 6 Department of Neurodegenerative Disease, UCL Institute of Neurology, London, UK; 7 UK Dementia Research Institute, London, UK; 8 Department of Psychiatry and Psychotherapy, Universitätsklinikum Erlangen and Friedrich-Alexander Universität Erlangen-Nürnberg, Erlangen, Germany; 9 Department of Neurodegeneration Diagnostics, Medical University of Bialystok, Bialystok, Poland; 10 Lab for Clinical Neurochemistry and Neurochemical Dementia Diagnostics, Department of Psychiatry and Psychotherapy, Universitätsklinikum Erlangen, Schwabachanlage 6, 91054 Erlangen, Germany

**Keywords:** Alzheimer’s Disease, Amyloidβ Peptides, Aβ_42/40_ ratio, Biomarkers, Cerebrospinal Fluid

## Abstract

The cerebrospinal fluid (CSF) biochemical markers (biomarkers) Amyloidβ 42 (Aβ_42_), total Tau (T-tau) and Tau phosphorylated at threonine 181 (P-tau_181_) have proven diagnostic accuracy for mild cognitive impairment and dementia due to Alzheimer’s Disease (AD). In an effort to improve the accuracy of an AD diagnosis, it is important to be able to distinguish between AD and other types of dementia (non-AD). The concentration ratio of Aβ_42_ to Aβ_40_ (Aβ_42/40_ Ratio) has been suggested to be superior to the concentration of Aβ_42_ alone when identifying patients with AD. This article reviews the available evidence on the use of the CSF Aβ_42/40_ ratio in the diagnosis of AD. Based on the body of evidence presented herein, it is the conclusion of the current working group that the CSF Aβ_42/40_ ratio, rather than the absolute value of CSF Aβ_42_, should be used when analysing CSF AD biomarkers to improve the percentage of appropriately diagnosed patients.

## Introduction

Alzheimer’s Disease (AD) is the most prevalent form of age-related dementia. The clinical manifestation of AD is generally preceded by a relatively symptom-free preclinical phase [1]. After the first clinical symptoms appear in the prodromal phase, patients transition clinically into mild cognitive impairment (MCI) [2], which eventually results in AD dementia (ADD) [3]. These phases are accompanied by biochemical changes in the brain that are reflected in cerebrospinal fluid (CSF) [4, 5]. Decreases in CSF concentrations of amyloid-beta 42 (Aβ_42_) (a marker of amyloidosis) and elevations in tau species (markers of axonal damage and neurofibrillary tangles) are well-established as biomarkers useful for AD diagnosis [6, 7]. Importantly, analysis of these CSF biochemical markers (biomarkers) for AD has been shown to predict conversion from MCI to ADD with accuracies of > 80% [8–10].

Depending on their age, approximately 30–50% of patients with MCI will develop ADD within 5 years [11]. Therefore, early diagnosis is essential to enable appropriate counselling to take place, as well as for planning treatment and care. In addition, the possibility of making an early diagnosis, prior to the appearance of symptoms, is essential for the clinical evaluation of novel, potentially disease-modifying drugs for the treatment of AD.

In an effort to improve the accuracy of an AD diagnosis, it is important to be able to distinguish between AD and other types of dementia (non-AD) that are not characterized by amyloid pathology. Although the concentration of another amyloid peptide species, Aβ_40_, has been reported to be unaltered in AD, the concentration ratio of Aβ_42_ to Aβ_40_ (Aβ_42/40_ ratio) has been suggested to be superior to the concentration of Aβ_42_ alone in discriminating patients with AD [12, 13]. To date, there has been a lack of comprehensive reviews on the applicability of the CSF Aβ_42/40_ ratio in AD diagnosis.

Thus, the aim of the current article was to review the available evidence on the use of the CSF Aβ_42/40_ ratio in the (i) differential diagnosis of AD dementia vs non-AD dementias and (ii) prediction of subsequent development of AD dementia in cases with MCI, and to discuss its value in comparison to other CSF biomarkers and compared to other diagnostic modalities, such as Aβ positron emission tomography (Aβ-PET). In addition, the effects of non-AD pathologies and pre-analytical handling on the various CSF biomarkers were also discussed. In order to achieve these goals, a working group was brought together to critically evaluate the evidence for use of the CSF Aβ_42/40_ ratio in the diagnosis of AD and a consensus paper was drafted reviewing the advantages and disadvantages surrounding the use of the CSF Aβ_42/40_ ratio.

This review addresses the advantages and disadvantages of the Aβ_42/40_ ratio to detect Aβ pathology, which is an approach to normalize the Aβ_42_ CSF concentration for the total Aβ CSF concentration (represented by the most abundant isoform, i.e. Aβ_40_). In contrast, this paper does not address approaches to interpret the overall pattern of the AD CSF biomarkers that combine results derived from the two distinct AD pathologies (amyloidosis and neurodegeneration) to form any kind of ‘ratios’.

## Methods

A group of experts in the field of AD biomarkers were brought together, and several meetings of this working group were conducted. During the meetings, discussions took place based both on evidence gathered from scientific publications and the experience of the group members, as experts in the field. Studies were selected to be included in this paper based on an independent review by at least two (in most cases by all) co-authors of the report, with MEDLINE database as the primary source of the studies. Searches were conducted using keywords, such as ‘Aβ_42/40_’ and ‘Aβ_40_’ and excluding those, whose primary scope was not the role of the Aβ_42/40_ ratio in AD diagnostics. The results of the discussions and the evidence gathered during the meetings are presented in this paper.

## Results

### Aβ_42_ versus the Aβ_42/40_ ratio

#### Comparison of the diagnostic accuracy in the context of use of differential diagnostics when discriminating AD from other neurodegenerative disorders

Due to similar clinical manifestations and overlapping brain pathologies, differentiation of AD from other neurodegenerative disorders may prove difficult even with the aid of biomarkers. For example, the symptoms and biomarker patterns observed in patients with dementia with Lewy bodies (DLB) or subcortical vascular dementia (VaD) sometimes closely resemble those of AD, which makes differential diagnosis difficult and decreases the diagnostic accuracy of the core CSF AD biomarkers, especially in the early stages of the disease. Therefore, evidence was gathered on whether adding the CSF Aβ_42/40_ ratio to the existing panel of biomarkers could improve the accuracy of the differential diagnosis of AD from other dementia disorders. Here we provide details of 16 studies that have compared the diagnostic accuracy of CSF biomarkers to diagnose ADD versus non-ADDs. These studies demonstrate the usefulness of the CSF Aβ_42/40_ ratio for the diagnosis of AD in patients with dementia. Studies with relevant data are also summarized in Table 1.

**Table 1:** CSF biomarkers to distinguish cases with ADD from cases with non-ADD

Study	Number of AD patients	Number of non-AD patients	Number of control patients	CSF biomarkers	Optimal cut-off*	Sensitivity % (95% CI)**	Specificity % (95% CI)**	AUC (95% CI)	SL (*p* value)^#^
Shoji et al. [12]	55	68	34	Aβ_42_	158.6 fmol/mL	–	–	–	–
				Aβ_42/40_ ratio^##^	0.078^##^	51	82	–	NP
Lewczuk et al. [13]	22	11	35	Aβ_42_	550 pg/mL	100	80	0.923	–
				Aβ_42/40_ ratio	9.75	95.2	88.4	0.944	NP
Spies et al. [22]	69	69	47	AD vs controls					
		16 DLB		Aβ_42_	–	93	87	0.949	–
		27 FTD		Aβ_42/40_ ratio	–	93	87	0.947	NP
		26 VaD		AD vs non-AD					
				Aβ_42_	–	83	74	0.811	NP
				Aβ_42/40_ ratio	–	85	85	0.903	
Hertze et al. [32]	94	166 (MCI) 29 (DD)	38	AD vs controls					
				Aβ_42 MSD_	< 523	73	89	0.88 (0.82–0.93)	–
				Aβ_42 MSD_/_40_ ratio	< 0.069	93	86	0.91 (0.86–0.95)	NP
				Aβ_42 MSD_/_38_ ratio	< 0.37	87	82	0.89 (0.83–0.93)	NP
				MCI-AD vs MCI					
				Aβ_42 MSD_	< 523	67	71	0.73 (0.66–0.80)	–
				Aβ_42 MSD_/_40_ ratio	< 0.069	85	71	0.86 (0.79–0.91)	NP
				Aβ_42 MSD_/_38_ ratio	< 0.37	88	71	0.85 (0.79–0.91)	NP
Gabelle et al. [14]	52	34	42	AD vs FTD					
				Aβ_42_	> 464	79	62	0.75	–
				Aβ_42/40_ ratio	≤11.1	79	76	0.85	n.s.
				Aβ_42_/_38_ ratio	≤2.00	88	86	0.87	n.s.
Slaets et al. [16]	80 69 (NP) 11 (AD+CVD)	75 24 DLB (15 NP) 29 FTD (12 NP) 22 VaD (11 NP)	30	Aβ_42_	517 pg/mL	81	59	0.747 (0.670–0.827)	–
				Aβ_42/40_ ratio	0.057	81	60	0.749 (0.673–0.826)	NP
Nutu et al. [17]	48	127 43 PD 33 PDD							
		51 DLB	107	AD vs control					
				Aβ_42_	444 ng/L	94	72	0.871 (0.811–0.930)	-NP
				Aβ_42/40_ ratio	0.125	92	79	0.871 (0.801–0.933)	
				AD vs PDD					
				Aβ_42_	449 ng/L	94	61	0.805 (0.704–0.905)	–
				Aβ_42_/_40_ ratio	0.150	90	81	0.910 (0.844–0.976)	NP
				AD vs DLB					
				Aβ_42_	387 ng/L	88	41	0.675 (0.570–0.780)	–
				Aβ_42/40_ ratio	0.115	90	57	0.759 (0.664–0.853)	NP
Baldeiras et al. [18]				AD vs controls					
				Aβ_42_	534 pg/mL	82	74	0.818	–
				Aβ_40/42_ ratio	8.3	59	81	0.719	NP
				AD vs FTD					
				Aβ_42_	538 pg/mL	70	82	0.791	–
				Aβ_40/42_ ratio	5.4	59	87	0.778	NP
Dumurgier et al. [19]	367 (AD+ non-AD)	–	0	AD vs non-AD					
				Aβ_42_	737–836 pg/mL	78	79	0.81	–
				Aβ_42/40_ ratio	0.050–0.082	73	78	0.81	NP
Struyfs et al. [23]	100	50	50	AD vs controls					
	50 (AD)	17 (DLB)		Aβ_42_	< 722 pg/mL	98.0	74.0	0.874	–
	50 (MCI-AD)	17 (FTD)		Aβ_42/40_ ratio	< 0.1099	85.7	78.0	0.881	NP
		16 (VaD)		Aβ_42/38_ ratio	< 0.269	81.6	82.0	0.858	NP
				AD vs non-AD					
				Aβ_42_	< 694 pg/mL	95.9	40.0	0.686	–
				Aβ_42/40_ ratio	< 0.1215	93.9	50.0	0.782	NP
				Aβ_42/38_ ratio	< 0.2730	81.6	68.0	0.804	NP
Bousiges et al. [25]	70	55	15	Pro-AD vs pro-DLB					
	31 (AD-d)	20 (DLB-d)		Aβ_42_	≤ 730 ng/L	84.6	71.4	0.84 (0.74–0.92)	–
	39 (pro-AD)	35 (pro-DLB)		Aβ_42/40_ ratio	≤ 0.0529	88.9	100	0.95 (0.83–0.99)	NP
				AD-d vs DLB-d					
				Aβ_42_	≤ 504 ng/L	67.7	80	0.77 (0.63–0.88)	–
				Aβ_42/40_ ratio	≤ 0.0799	92.3	88.9	0.86 (0.64–0.97)	NP
Janelidze et al. [24]	Cohort 2	Cohort 2	Cohort 2	AD vs MCI					
	75 (AD)	62 (MCI)	53	Aβ_42_	–	–	–	0.817 (0.743–0.890)	–
	35 (MCI-AD)	34 (VaD)	Cohort 3	Aβ_42/40_ ratio	–	–	–	0.879 (0.823–0.936)	< 0.028
	Cohort 3	47 (DLB/PDD)	328	Aβ_42/38_ ratio	–	–	–	0.856 (0.790–0.923)	< 0.222
	137	33 (FTD)		AD vs DLB/PDD					
		Cohort 3		Aβ_42_	–	–	–	0.583 (0.476–0.690)	–
		35 (DLB/PDD)		Aβ_42/40_ ratio	–	–	–	0.792 (0.707–0.877)	< 0.001
		128 (PD)		Aβ_42/38_ ratio	–	–	–	0.796 (0.710–0.883)	< 0.001
				AD vs VaD					
				Aβ_42_	–	–	–	0.698 (0.580–0.816)	–
				Aβ_42/40_ ratio	–	–	–	0.880 (0.814–0.946)	< 0.001
				Aβ_42/38_ ratio	–	–	–	0.860 (0.786–0.935)	< 0.001
				AD vs non-AD					
				Aβ_42_	–	–	–	0.720 (0.651–0.788)	–
				Aβ_42/40_ ratio	–	–	–	0.863 (0.813–0.912)	< 0.001
				Aβ_42/38_ ratio	–	–	–	0.863 (0.813–0.913)	< 0.001
Lehmann et al. [26]	342	562	0	AD vs non-AD					
	Cohort 1	Cohort 1		Cohort 1					
	124	276		Aβ_42_	500 pg/mL	–	–	0.78 (0.734–0.818)	–
	Cohort 2	Cohort 2		Aβ_42/40_ ratio	0.1	–	–	0.90 (0.865–0.926)	< 0.0001
	218	286		Cohort 2					
				Aβ_42_	700 pg/mL	–	–	0.60 (0.553–0.641)	–
				Aβ_42/40_ ratio	0.05	–	–	0.77 (0.728–0.803)	< 0.0001

*Optimal cut-offs were created using different statistical approaches—please see original articles for details. **Sensitivity and specificity are a function of the cut-off, and the cut-offs were calculated in different ways; therefore, they are not clearly comparable across different articles. ^#^Significance levels (*p* values) of the AUC values are comparisons of the Aβ isoform ratios vs Aβ_42_ alone. ^##^Note that the ratio in the original article is inversed, but for consistency, the Aβ_42/40_ ratio has been calculated for this table. *Aβ* Amyloid beta, *AD* Alzheimer’s Disease, *ADD* AD dementia, *Ad-d* demented AD patients, *AUC* area under curve, *CJD* Creutzfeldt-Jakob Disease, *CSF* cerebrospinal fluid, *DLB* dementia with Lewy bodies, *DLB-d* demented DLB patients, *FTD* frontotemporal dementia, *MCI* mild cognitive impairment, *MCI-AD* MCI that subsequently developed ADD or MCI due to AD, *NP* neuropathologically confirmed, *NP* not provided, *NPH* normal pressure hydrocephalus, *n.s* not significant, *PD* Parkinson’s Disease, *PDD* Parkinson’s Disease dementia, *pro-AD* prodromal-AD patients, *pro-DLB* prodromal-DLB patients, *PsD* psychiatric disorders, *SL* significance level, *VaD* vascular dementia

In a study of patients with ADD, normal controls, patients with non-ADD and patients with other neurological diseases, Shoji et al. [12] found that the ADD group had a significantly higher level of tau than the normal control group (*p* <  0.001), but Aβ_40_ levels did not show any significant differences between the groups. The reduction of Aβ_42_ levels in AD also resulted in a significant increase in the Aβ_40/42_ ratio (note that the ratio reported in this study has Aβ_40_ in the numerator, in contrast to most of the other studies summarized in the current paper) as an improved marker. The authors therefore concluded that the Aβ ratio is another important marker for AD.

Lewczuk et al. [13] measured concentrations of Aβ_42_, Aβ_40_ and total tau (T-tau) in order to compare their accuracy in discriminating patients with ADD, non-ADD and control subjects. The results showed that concentrations of Aβ_42_ were decreased (*p* <  0.001) and of T-tau were increased (*p* <  0.001) in ADD patients, while Aβ_40_ concentrations did not differ significantly among the groups. For all groups when the Aβ_42/40_ ratio was used, more patients were classified correctly, compared to when the Aβ_42_ concentration alone was used (94 vs 86.7% when comparing ADD to controls, 90 vs 85% when comparing ADD to non-ADD and 90.8 vs 87% when comparing ADD to non-ADD plus controls). The improvement of the diagnostic accuracy reported in this study was not significant, probably due to the small numbers of subjects and a clear ceiling effect (a relatively high number of patients were already correctly classified using Aβ_42_ alone).

Gabelle et al. [14] evaluated the value of individual and combined measurements of CSF biomarkers. They found that both Aβ_40/42_ and Aβ_38/42_ ratios were significantly altered in AD. They also found that the Aβ_40/38_ ratio was the only one that differentiated clearly control subjects from FTD subjects, while not being significant between AD and FTD. In the ROC curves, they found that for FTD versus AD diagnosis, the best AUCs for amyloid biomarkers were the Aβ_38/42_ ratio and the Innogenetics Aβ/Tau index (IATI) (AUCs = 0.87). However, the Aβ_40/42_ ratio or Aβ_42_ alone had very close and statistically undifferentiated AUC values. The authors concluded that the Aβ_38/42_, Aβ_40/42_ and the IATI ratios were also better than individual biomarkers to identify AD therefore justifying their clinical relevance.

In another study carried out by Wiltfang et al. [15], the authors found that alterations of Aβ_42_ concentrations might not only result from AD pathology but may also be related to total Aβ peptide concentrations. In such cases, healthy individuals with relatively low total Aβ might be misdiagnosed as having ‘pathologically low’ Aβ_42_ concentrations, and vice versa, AD subjects with high total Aβ might be misinterpreted as normal Aβ_42_ carriers. It was therefore concluded that the analysis of CSF Aβ_42_ alone (i.e. without Aβ_40_) might lead to misinterpretation of the neurochemical dementia diagnostics outcome in subjects with constitutively high or low concentrations of total Aβ peptides. Consequently, the authors conclude that the CSF Aβ_42/40_ ratio can possibly improve the reliability of the neurochemical dementia diagnosis.

A study by Slaets et al. [16] compared the use of different biomarkers for the diagnosis of AD. Addition of the CSF Aβ_42/40_ ratio to the existing panel of biomarkers, Aβ_42_, Aβ_40_ and tau phosphorylated at threonine 181 (P-tau_181_) was compared to the panel without the addition of the ratio. The results showed that the CSF Aβ_42/40_ ratio was significantly decreased in AD patients (0.043 ± 0.021) compared to non-AD patients (0.064 ± 0.027; *p* <  0.001) and controls (0.053 ± 0.023; *p* < 0.001). Following receiver operating characteristic (ROC) analysis, the optimal cut-offs discriminating the groups were defined as the values maximising the sum of the sensitivity and the specificity. Although the difference between the areas under the ROC curves (AUC) for Aβ_42_ and Aβ_42/40_ turned out to be insignificant, the diagnostic accuracy of the decision tree that contained Aβ_42_, Aβ_40_, P-tau_181_ and the Aβ_42/40_ ratio was significantly better than the diagnostic accuracy of the decision tree without Aβ_40_ and the Aβ_42/40_ ratio (80% vs 74%; *p* < 0.001). The authors concluded that there was no difference in CSF Aβ_40_ levels found between AD and non-AD patients, but that adding CSF Aβ_40_ and the CSF Aβ_42/40_ ratio to a biomarker-based decision tree, might have an added value for discriminating AD from non-AD patients in cases with intermediate CSF P-tau_181_ values.

Nutu et al. [17] evaluated whether the CSF Aβ_42/40_ ratio could be used for differentiating AD from DLB and Parkinson’s Disease Dementia (PDD). The primary finding of this study was that the CSF Aβ_42/40_ ratio increased discrimination of AD from PDD and DLB compared with either of the two Aβ biomarkers individually. Of note, in this study, Aβ_40_ was significantly higher in AD. Furthermore, the authors concluded that use of the Aβ_42/40_ ratio could improve the differentiation of AD from PDD and DLB.

In a study by Baldeiras et al. [18], the added value of another CSF Aβ-peptide (Aβ_40_), along with the core CSF markers T-tau, P-tau_181_ and Aβ_42_, in the discrimination between two large dementia groups of FTD (*n* = 107), AD (*n* = 107) and non-demented subjects (*n* = 33) was evaluated. The authors found that their data ‘taken together’ indicated that ‘although CSF Aβ_40_ has no added value in the distinction between AD and FTD patients, it might be useful in the discrimination between AD and FTD patients from non-demented controls, and therefore could be considered in patients diagnostic work-up’.

In a prospective study of subjects with cognitive disorders at three French memory centres (Paris-North, Lille and Montpellier; the PLM study), Dumurgier et al. [19] assessed whether the use of the Aβ_42/40_ ratio would reduce the frequency of indeterminate CSF profiles. They found that, on the basis of local optimum cut-offs for Aβ_42_ and P-tau_181_, 22% of patients had indeterminate CSF profiles. The systematic use of the Aβ_42/40_ ratio instead of Aβ_42_ levels alone decreased the number of indeterminate profiles (17%; *p* = 0.03), but it failed to improve the classification of subjects (NRI = − 2.1%; *p* = 0.64). In contrast, use of the Aβ_42/40_ ratio instead of Aβ_42_ levels alone in patients with a discrepancy between P-tau_181_ and Aβ_42_ led to a reduction by half of the number of indeterminate profiles (10%; *p* < 0.001) and was also in agreement with clinician diagnosis (NRI = 10.5%; *p* = 0.003). The authors therefore concluded that in patients with a discrepancy between CSF P-tau_181_ and CSF Aβ_42_, the assessment of the Aβ_42/40_ ratio led to a reliable biological conclusion in over 50% of cases that agreed with a clinician’s diagnosis.

Sauvee et al. [20] investigated whether the CSF Aβ_42/40_ ratio could be used to improve the accuracy of diagnostically relevant conclusions in patients with ambiguous CSF Aβ_42_ or tau results. They found that one third of the biomarker profiles of patients with atypical dementia were ambiguous. The addition of the CSF Aβ_42/40_ ratio increased the proportion of interpretable profiles from 69 to 87%, without changing the conclusion when the usual biomarkers (Aβ_42_ and P-tau_181_) were concordant. The authors therefore concluded that their results support the use of the Aβ_42/40_ ratio in addition to the usual CSF AD biomarkers for patients with ambiguous profiles. They added that this method could be specifically directed to this population (i.e. patients with ambiguous results) in order to improve the level of certainty for clinical routine practice.

Lewczuk et al. [21] also compared the diagnostic accuracies of the CSF Aβ_42/40_ ratio and CSF Aβ_42_ alone. Analysis of Aβ_42_ gave a sensitivity and specificity of 69.3% and 88.9%, respectively, whereas the Aβ_42/40_ ratio showed significantly improved performance with sensitivity and specificity of 93.3% and 100%, respectively. Thus, the authors concluded that the CSF Aβ_42/40_ ratio concentration shows significantly better diagnostic performance compared to the CSF Aβ_42_ concentration alone. It should be noted, however, that this study must not be interpreted as providing absolute values of the diagnostic accuracies, but their relative comparison.

In another study including various CSF biomarkers, Spies et al. [22] investigated the CSF Aβ_42/40_ ratio under the assumption that Aβ_40_ closely represents the total cerebral Aβ load. They found that the Aβ_42/40_ ratio improves differentiation of AD patients from VaD, DLB and non-ADD patients when compared to Aβ_42_ alone. Furthermore, they found that the Aβ_42/40_ ratio is a more easily interpretable alternative to the combination of Aβ_42_, P-tau_181_ and T-tau when differentiating AD from either frontotemporal dementia (FTD) or other non-ADDs. Since they found different Aβ_40_ concentrations in the various dementia groups, the authors also added that it can be debated if the Aβ_42/40_ ratio is a good representation of the Aβ_42_ fraction of the total Aβ load and thus eliminates inter-individual differences in total Aβ concentrations.

A study of patients with AAD, non-ADDs (DLB, FTD, VaD), MCI due to AD and non-demented controls found that the CSF Aβ_42/40_ ratio improved the diagnostic performance of Aβ_42_ in most differential diagnostic situations. Struyfs et al. [23] also found that the Aβ_42/40_ ratio was the best biomarker to distinguish between AD-MCI and FTD.

Similarly, Janelidze et al. [24] also found that the CSF Aβ_42/40_ ratio, as well as the CSF Aβ_42/38_ ratio, was ‘superior biomarkers of AD pathology compared with Aβ_42_ alone’. Using three commercially available CSF biomarker immunoassays, this study found that the CSF Aβ_42/40_ and Aβ_42/38_ ratios improved differentiation of AD from non-ADs, especially when separating AD from DLB/PDD and VaD.

The authors point to several potential explanations for the improved accuracy when using the CSF Aβ_42/40_ and Aβ_42/38_ ratios instead of Aβ_42_. They suggest that it might be that subcortical pathologies not specific to AD, such as WMLs and alpha-synuclein pathology, cause reduced levels of all CSF Aβ species, including Aβ_42_. A second explanation for the improved diagnostic accuracy of the Aβ_42/40_ and Aβ_42/38_ ratios could be that differences in the overall production and clearance of Aβ probably contribute to inter-individual variability in total CSF Aβ levels. This is supported by the present finding that in CSF Aβ_42_ correlates with Aβ_38_ and Aβ_40_ even in healthy controls. Consequently, when detecting Aβ_42_ brain pathology with CSF Aβ_42_, using ratios to Aβ_40_ or Aβ_38_ might correct for inter-individual differences in total Aβ levels.

In another study, which evaluated the differential diagnosis of DLB and AD, once again, the CSF Aβ_42/40_ ratio was found to aid diagnosis. The study by Bousiges et al. [25] found that the Aβ_42/40_ ratio remained unchanged in DLB patients between the prodromal and demented stages, contrary to what was observed in AD. The Aβ_42/40_ ratio therefore makes it possible to distinguish between the two pathologies ‘at a time when the differential diagnosis is difficult’.

Finally, in a study by Lehmann et al. [26], the Aβ_42/40_ ratio was added to a previously described PLM scale (Paris-Lille-Montpellier study), which combines Aβ_42_, T-tau and P-tau_181_ biomarkers, in order to evaluate an optimized PLM_R_ scale (PLM ratio scale). Nine hundred and four participants (342 AD and 562 non-AD) were studied, in two chronologically different cohorts (400 Mtp-1 and 504 Mtp-2). For AD patients, the mean CSF Aβ_42_ and CSF Aβ_42/40_ ratio was 553 ± 216 pg/mL and 0.069 ± 0.022 pg/mL in Mtp-1 and 702 ± 335 pg/mL and 0.045 ± 0.020 pg/mL in Mtp-2. The distribution of AD and non-AD differed between the PLM and the PLM_R_ scales (*p* < 0.0001). The percentage AD well-classified (class 3) increased with PLM_R_ from 38 to 83% in Mpt-1 and from 33 to 53% in Mpt-2. A sharp reduction of the discordant profiles going from 34 to 16.3% and from 37.5 to 19.8%, for Mtp-1 and Mtp-2 respectively, was also observed. The authors concluded that the integration of the Aβ_42/40_ ratio in the PLM_R_ scale resulted in an easy-to-use tool which reduced the discrepancies in biologically doubtful cases and increased the confidence in the diagnosis.

In order to try to assess what is the overall impact on diagnosis, an estimation was made of what the actual percentage of patients that are misdiagnosed by Aβ_42_ alone and that become correctly classified with the Aβ_42/40_ ratio. Assuming normal distribution of Aβ_40_ across the population of interest [15], a very conservative estimation is that neglecting Aβ_40_ (which is equivalent to applying Aβ_42_ as the sole CSF biomarker of amyloidosis) leads to misdiagnoses of ca. 5–10% of cases. This is further confirmed by Baldeiras et al. [27], who found an increase in the proportion of interpretable CSF profiles from 61 to 75% (i.e. ca. 20% of the baseline value) of the MCI patients. Also, Dorey et al. [28] report that determining CSF Aβ_40_ concentrations corrected diagnosis in AD patients with non-pathological CSF Aβ_42_ levels in 76.2% of cases using the CSF Aβ_42/40_ ratio.

In summary, the accumulation of evidence clearly points to the usefulness of the CSF Aβ_42/40_ ratio for the diagnosis of AD in patients with dementia. The CSF Aβ_42/40_ ratio is also better than CSF Aβ_42_ alone at distinguishing AD dementia from non-AD dementias, not only from controls. The evidence therefore strongly suggests that the CSF Aβ_42/40_ ratio, rather than CSF Aβ_42_ alone, should be used in the clinical work-up of AD, as a way to improve the diagnostic accuracy for distinguishing ADD from other dementia disorders.

### Comparison of the diagnostic accuracy for predicting the development of ADD in patients with MCI

With disease-modifying therapies on the horizon, there is a need to be able to predict the risk of developing ADD before the onset of dementia, i.e. during the MCI and subjective cognitive decline (SCD) stages. The increased percentage of MCI subjects with pathologic CSF who convert to ADD, compared to those having normal CSF, is considered a very strong argument in favour of the use of CSF biomarkers as predictors of MCI to ADD conversion. Here we provide details of six studies that have compared the diagnostic accuracy of CSF biomarkers when predicting the development of ADD in patients with MCI. All of the studies show the added value of the CSF Aβ_42/40_ ratio in accurately predicting progression to ADD. Studies with relevant data are also summarized in Table 2.

**Table 2:** CSF biomarkers for predicting the development of AD in patients with MCI

Study	Number of AD patients	Number of MCI/ non-AD patients	Number of control patients	CSF biomarkers	Optimal cut-off*	Sensitivity % (95% CI)**	Specificity % (95% CI)**	AUC (95% CI)	SL (*p* value)^#^
Hansson et al. [31]	0	137 (MCI)	0	Aβ_42_	0.64 ng/mL	93 (82–98)	53 (41–64)	0.77 (0.69–0.84)	–
				Aβ_42/40_ ratio	0.95	87 (76–95)	78 (67–86)	0.87 (0.80–0.92)	< 0.05
Hertze et al. [32]	94	166 (MCI) 29	38	MCI-AD vs MCI					
		(DD)		Aβ_42 MSD_	< 523	67	71	0.73 (0.66–0.80)	–
				Aβ_42 MSD_/_40_ ratio	< 0.069	85	71	0.86 (0.79–0.91)	NP
				Aβ_42 MSD_/_38_ ratio	< 0.37	88	71	0.85 (0.79–0.91)	NP
Parnetti et al. [33]	28 (AD) 32	58 (MCI-MCI)	0	MCI-AD vs MCI-MCI					
	(MCI-AD)	28 (OND)		Aβ_42_	420.5 pg/mL	56 (38–74)	96 (88–99)	0.85	–
				Aβ_42/40_ ratio	5.3	71 (48–89)	92 (79–98)	0.82	NP
				AD vs non-AD (OND)					
				Aβ_42_	500.0 pg/mL	63 (42–81)	79 (59–92)	0.70	–
				Aβ_42/40_ ratio	5.0	74 (54–89)	79 (59–92)	0.78	NP
Lewczuk et al. [21]	75 (AD-MCI)	0	45	Aβ_42_	691 pg/mL	69.3	88.9	0.895 (0.819–0.946)	–
				Aβ_42/40_ ratio	0.06	93.3	100	0.970 (0.916–0.993)	< 0.0001
Baldeiras et al. [27]	168	197 (MCI)	66	Aβ_42_	585 pg/mL	82	83	0.882 (0.837–0.927)	–
				Aβ_42/40_ ratio	0.068	79	86	0.874 (0.827–0.921)	n.s.
Frölich et al. [34]	0	115 (MCI)	0	Aβ_42_	< 600 pg/mL	74	64	0.68	–
				Aβ_42/40_ ratio	–	59	75	0.66	NP

*Optimal cut-offs were created using different statistical approaches—please see original articles for details.**Sensitivity and specificity are a function of the cut-off, and the cut-offs were calculated in different ways, therefore they are not clearly comparable across different articles. ^#^Significance levels (*p* values) of the AUC values are comparisons of the Aβ isoform ratios vs Aβ_42_ alone. *Aβ* Amyloid beta, *AD* Alzheimer’s Disease, *AD-MCI* early AD and MCI, *AUC* area under curve, *DD* depressive disorder, *MCI* mild cognitive impairment, *MCI-AD* mild cognitive impairment patients progressing to AD, *MCI-MCI* stable MCI patients, *MSD* Meso Scale Discovery assay, *NP* not provided, *n.s* not significant, *OND* other neurological diseases, *SL* significance level

In a study validating the previously introduced Erlangen Score interpretation algorithm [29], Lewczuk et al. compared three- and four-biomarker-based approaches in the German Competence Network Dementias cohort. They found that the score based on four biomarkers (i.e. including the Aβ_42/40_ ratio in addition to Aβ_42_) correlated better with the ratio of pre-dementia subjects having progressed to ADD than the score based on three biomarkers [30].

In a study by Hansson et al. [31], baseline levels of Aβ_40_ and Aβ_42_ in CSF were measured in a cohort of patients with MCI (*n* = 137) in relation to the final diagnosis after 4–6 years of follow-up. The Aβ_42_ concentration at baseline and the Aβ_42/40_ ratio were significantly decreased in MCI patients who developed ADD compared to cognitively stable MCI patients and MCI patients who developed other forms of dementia (*p* < 0.001). The baseline levels of Aβ_40_ were similar in all MCI groups but correlated with change in Mini-Mental State Examination scores in converters to ADD. The Aβ_42/40_ ratio was superior to Aβ_42_ concentration with regard to identifying incipient AD in MCI (*p* < 0.05). The authors concluded that the data provides support for the view that amyloid precursor protein metabolism is disturbed in early sporadic AD and points to the usefulness of the Aβ_42/40_ ratio as a predictive biomarker for AD.

A study by Hertze et al. [32] investigated the diagnostic accuracy of CSF biomarkers to predict the development of ADD within 5 years in patients with MCI. The results showed that the predictive values of the Aβ_42/40_ and Aβ_42/38_ ratios were higher compared to that of Aβ_42_ alone (*p* < 0.01) when using the electrochemiluminescence technology of the Meso Scale Discovery (MSD) platform to quantify amyloid in MCI patients. However, Aβ_42_ quantified with a bead-based multiplexing (xMAP) technology performed as well as the MSD Aβ_42/40_ ratio.

A 4-year follow-up study carried out by Parnetti et al. [33] measured CSF Aβ_40_, Aβ_42_, T-tau and P-tau_181_ in patients with AD, stable MCI (MCI-MCI) and MCI evolving into ADD (MCI-AD) in order to evaluate the power of each biomarker and/or their combination in predicting AD progression. Although they found that inclusion of the Aβ_42/40_ ratio instead of Aβ_42_ alone did not improve the prediction power of the model in the multivariate analysis, when univariate statistics were employed, they found that the Aβ_42/40_ ratio had an increased sensitivity with respect to Aβ_42_.

In a recently published paper, Baldeiras et al. [27] showed that replacing Aβ_42_ by the Aβ_42/40_ ratio resulted in a significant increase in the proportion of interpretable biological profiles (from 61 to 75%, *p* = 0.001) of MCI patients, due to a reduction by half in the number of suspected non-Alzheimer pathophysiology cases and an increase in the proportion of the high-AD-likelihood subgroup. In their study, the risk of progression to ADD was highest in the ‘high-likelihood’ group and increased when the Aβ_42/40_ ratio, instead of Aβ_42_, combined with T-tau and P-tau_181_ was used for biomarker-based categorization.

Frölich et al. [34] investigated whether the progression of MCI to AD dementia can be predicted by cognitive, neuroimaging and CSF markers. They studied 115 complete datasets from MCI patients of the ‘Dementia Competence Network’, a German multi-centre cohort study with annual follow-up up to 3 years. They hypothesized that since most biomarkers reveal complementary information, a combination of biomarkers may increase the predictive power. Their results showed that two- to four-parameter combinations of the eight predictor/biomarker indices (MMSE, CDR-sb, CERAD-DR, HCV, Aβ_42_, Aβ_42/40_, T-tau and P-tau_181_) were numerically superior over the performance of a single biomarker index in predicting MCI subjects who progressed to AD. In this dataset, however, the Aβ42/Aβ40 ratio was not consistently superior to Aβ_42_ alone for predicting AD dementia in MCI patients.

Together these reports provide evidence that clearly demonstrates the added value of the CSF Aβ_42/40_ ratio in accurately predicting progression to ADD.

In the last years, studies have also been published applying the CSF Aβ_38_ concentration as the reference isomer for normalization of the Aβ_42_ concentration (in the form of Aβ_42/38_ ratio). However, it needs to be stressed that neither the results of these studies, nor the physiological rationale behind using Aβ_38_ instead of Aβ_40_ is convincing enough to replace Aβ_40_.

### Comparison of the diagnostic accuracy of the CSF biomarkers and Aβ-PET

CSF Aβ_42_ and Aβ-PET have both been found to correlate highly with brain biopsy findings [35, 36]. Decreased concentrations of CSF Aβ_42_ and increased retention of amyloid tracers in the brain on PET are considered the earliest biomarkers of AD, although some evidence suggests that the alterations measurable in the CSF occur earlier [43]. A potential advantage of Aβ-PET over CSF Aβ_42_ as an early diagnostic marker is the possibility to detect regional Aβ depositions that might occur before the global neocortical signal becomes pathologic. On the other hand, analysis of CSF offers a quantitative result of the net effect of the biomarkers. Running costs of CSF analysis are 10- to 15-fold lower than those for Aβ-PET (total costs for lumbar puncture and analysis of four CSF biomarkers in Germany do not exceed €200). CSF analysis is also more accessible to patients, it does not require the patient and caregiver to travel to a distant centre equipped with PET facilities and it enables simultaneous analysis of dozens of biomarkers in one sample volume, including biomarkers of neurodegeneration, neuroinflammation and others [37–39]. In contrast to neuroimaging modalities, a CSF-based approach enables aliquoting and storage of a sample’s volume for further analyses in the future, also in other laboratories.

Finally, although lumbar puncture is frequently regarded as an invasive procedure, serious complications are extremely rare, with the most common, headache, only being observed in 2% of the elderly population and being easily treated with simple analgesia [40]. On the other hand, it is questionable to consider injection of radioactive tracers, designed to target brain tissue, as a ‘non-invasive’ procedure. Here we provide details of five studies that have compared the diagnostic accuracy of CSF biomarkers and Aβ-PET, all showing that both methods can identify early AD with high accuracy. Studies with relevant data are also summarized in Table 3.

**Table 3:** Studies comparing CSF biomarkers and Aβ-PET

Study	Number of AD patients	Number of MCI/ non-AD patients	Number of control patients	CSF biomarkers/ PET	Optimal cut-off*	Sensitivity% (95% CI)**	Specificity% (95% CI)**	AUC (95% CI)	SL (*p* value)^#^
Mattsson et al. [42]	121	68 (SCD)	161	Aβ_42_	< 192 ng/L	–	–	–	–
		419 (MCI)		Aβ_42/40_ ratio		–	–		NP
				Florbetapir PET	> 1.11 SUV	–	–	–	NP
Janelidze et al. [24]	Cohort 1	Cohort 1	Cohort 1	Euroimmun					
	0	215 (MCI)	0	Aβ_42_	< 507.5 pg/mL	83.2	83.3	0.894 (0.850–0.937)	–
				Aβ_42/40_ ratio	< 0.10	97.2	88.0	0.954 (0.923–0.986)	0.008
				Aβ_42/38_ ratio	< 0.29	92.5	88.9	0.943 (0.911–0.975)	0.007
				MSD					
				Aβ_42_	< 495.9 pg/mL	85.0	88.9	0.916 (0.876–0.956)	–
				Aβ_42/40_ ratio	< 0.09	95.3	95.4	0.975 (0.952–0.998)	< 0.001
				Aβ_42/38_ ratio	< 0.17	97.2	91.7	0.964 (0.935–0.992)	0.007
				Quanterix					
				Aβ_42_	< 1742 pg/mL	73.3	77.5	0.810 (0.707–0.913)	–
				Aβ_42/40_ ratio	< 0.16	90.0	90.0	0.912 (0.834–0.991)	0.002
Lewczuk et al. [41]	0	199 CN &	0	Aβ_42_	735 pg/mL	81.6 (68.0–91.2)	72.7 (64.8–79.6)	0.814 (0.752–0.865)	–
		abnormal (150 PET−/ 49 PET+)		Aβ_42/40_ ratio	0.05	95.9 (86.0–99.5)	88.0 (81.7–92.7)	0.936 (0.892–0.966)	< 0.001
Janelidze et al. [44]	0	262 (MCI/SCD)	0	Innotest					
				Aβ_42_	≤ 548 pg/mL	96	82	0.92 (0.89–0.95)	–
				Aβ_42/40_ ratio	≤ 0.06	91	82	0.92 (0.88–0.95)	NP
				Modified Innotest					
				Aβ_42_	≤ 1091 pg/mL	92	74	0.87 (0.83–0.91)	–
				Aβ_42/40_ ratio	≤ 0.12	92	87	0.93 (0.90–0.96)	≤ 0.01
				Euroimmun					
				Aβ_42_	≤ 449 pg/	82	80	0.88	–
				Aβ_42/40_ ratio	mL ≤ 0.10	93	88	(0.84–0.92) 0.93 (0.90–0.96)	≤ 0.01
				MSD					
				Aβ_42_	≤ 506 pg/mL	94	76	0.89 (0.85–0.93)	–
				Aβ_42/40_ ratio	≤ 0.08	96	89	0.95 (0.93–0.98)	≤ 0.001
Schindler et al. [46]	0	22 (MCI)	176 (CN)	Aβ_42_	< 1.098 pg/mL	–	–	0.85 (0.80–0.90)	–
		(~ 25% PiB+)	(~ 25% PiB+)	Aβ_42/40_ ratio	< 0.075	–	–	0.93 (0.89–0.96)	NP

*Optimal cut-offs were created using different statistical approaches—please see original articles for details. **Sensitivity and specificity are a function of the cut-off, and the cut-offs were calculated in different ways, therefore they are not clearly comparable across different articles. ^#^Significance levels (*p* values) of the AUC values are comparisons of the Aβ isoform ratios vs Aβ_42_ alone. *Aβ* amyloid beta, *AD* Alzheimer’s Disease, *ADAS-cog* Alzheimer’s Disease Assessment Scale-cognitive subscale, *AUC* area under curve, *cen* centiloid, *CN* cognitively normal, *CU* centiloid units, *DLB* dementia with Lewy bodies, *FTD* frontotemporal dementia, *MCI* mild cognitive impairment, *MCI-AD* MCI that subsequently developed AD dementia, *MSD* Meso Scale Discovery, *NP* not provided, *n.s* not significant, *PD* Parkinson’s Disease, *PDD* Parkinson’s Disease with dementia, PET, positron emission tomography, *MS-RMP* mass spectrometry-based candidate reference measurement procedure, *PiB* carbon-11 labelled thioflavin-T derivative, Pittsburgh compound B, *SCD* subjective cognitive decline, *SL* significance level, *SUV* standardized uptake value, *VaD* subcortical vascular dementia, *vis* visual

In Cohort 1 of a study carried out by Janelidze et al. [24], which included 215 MCI patients, a discrepancy between CSF Aβ_42_ and Aβ-PET status was observed. In fact, they found that 10–20% of healthy individuals and MCI patients showed a mismatch in CSF Aβ_42_ and Aβ-PET status. In their study, they found that the CSF Aβ_42/40_ and Aβ_42/38_ ratios better predict abnormal cortical amyloid deposition (visualized with PET) compared with Aβ_42_. The ratios increased the classification performance both for patients who were falsely classified as positive (by low CSF Aβ_42_) and for patients who were falsely classified as negative (by high CSF Aβ_42_).

One possible explanation proposed by the authors for the improved concordance between Aβ-PET and CSF Aβ, when using the Aβ_42/40_ and Aβ_42/38_ ratios instead of Aβ_42_, was that subcortical pathologies not specific to AD could cause reduced levels of all CSF Aβ species, including Aβ_42_, but not the ratios. For example, in patients with MCI, low CSF Aβ_42_, Aβ_40_ and Aβ_38_ were all linked to subcortical injury, including increased white matter lesions (WML) and enlarged lateral ventricles. The mechanisms underlying these associations are likely related to dysregulation in β amyloid precursor protein pathways with a general decline in the production of Aβ.

A study by Lewczuk et al. [41] reported an association between amyloid alterations reflected in the CSF with those in Aβ-PET in a cohort of 199 patients. The results showed that the CSF Aβ_42/40_ ratio corresponded better than Aβ_42_ with PET results, with a larger proportion of concordant cases (89.4% vs 74.9%, respectively, *p* < 0.001) and a larger area under the ROC curve (AUC 0.936 vs 0.814, respectively, *p* < 0.001) associated with the ratio. For both CSF biomarkers, the percentage of CSF-abnormal/PET-normal cases was larger than that of CSF-normal/PET abnormal cases. The authors concluded that the CSF Aβ_42/40_ ratio is superior to Aβ_42_ alone as a marker of amyloid-positivity by PET, which may be explained by the fact that the ratio compensates for general between-individual variations in CSF total Aβ concentrations. Furthermore, they speculated, that the fact that there was a higher proportion of subjects with pathologic CSF (as reflected by Aβ_42_ or Aβ_42/40_) and normal PET compared to those with pathologic PET and normal CSF might suggest that CSF reflects amyloidosis earlier than PET does. Similar conclusions based on earlier findings in CSF compared to PET were also made by Mattsson et al. and Palmqvist et al. [42, 43].

Janelidze et al. [44] studied the concordance between CSF Aβ_42_ levels measured using five different immunoassays [Innotest, Modified Innotest (increased antibody concentration and incubation time, and lower CSF volume), fully automated Lumipulse, Euroimmun and MSD assays) and visual read of Aβ-PET images in 262 patients with MCI or SCD. They found that although the accuracy to correlate with visual 18F-flutemetamol PET was decreased when using newer Aβ_42_ assays, this limitation is overcome by using the CSF Aβ_42/40_ ratio. They also found that the CSF Aβ_42/40_ ratio from the newer assays showed improved accuracy for detection of cortical Aβ fibrils as measured by PET. Moreover, the sensitivities and specificities of these newer assays were less influenced by moderate changes in the cut-offs when the CSF Aβ_42/40_ ratio was used, a finding that is important when samples will be analysed consecutively over time.

In another study analysing the agreement between data on cerebral amyloidosis, derived using Pittsburgh compound B (PiB) PET and immunosorbent assay of CSF Aβ_42_, Leuzy et al. [45] examined 243 subjects with normal cognition and patients with MCI. They found that agreement between PiB classification and MSD immunosorbent assay/mass spectrometry reference measurement procedure findings was further improved when using the CSF Aβ_42/40_ ratio.

A study by Schindler et al. [46] studied the relationship between CSF biomarkers, as measured by a novel automated immunoassay platform and Aβ-PET. They found that CSF biomarker ratios (T-tau/Aβ_42_, P-tau_181_/Aβ_42_ and Aβ_42/40_) best discriminated PiB-positive from PiB-negative individuals.

These reports show that both Aβ-PET and CSF biomarkers can identify early AD with high accuracy. However, several studies strongly suggest that Aβ alterations in the CSF occur earlier [42, 43]. Similarly, there is no convincing evidence that the spatial distribution of Aβ deposits in the brain tissue observed in PET deliver clinically relevant information [47]. In addition, the CSF Aβ_42/40_ ratio can better predict abnormal cortical amyloid deposition (visualized with PET) compared with Aβ_42_. This then leads to fewer patients being diagnosed as false positive (low CSF Aβ_42_) or false negative (high CSF Aβ_42_).

### Effects of non-AD pathologies on Aβ_42_, Aβ_40_ and the Aβ_42/40_ ratio, such as WMLs, PDD and DLB

In clinical practice, CSF biomarker analyses are often carried out in patients with atypical or mixed presentation of dementia. This makes the diagnosis complex and highlights the importance of being able to discriminate AD from other neurodegenerative processes such as FTD, WMLs, VaD, DLB, PDD and cerebral amyloid angiopathy [5].

Selnes et al. [48] studied the effects of cerebrovascular disease on amyloid precursor protein metabolites in CSF in 37 patients with SCD or MCI without stroke, and 26 after acute stroke. They found that CSF levels of Aβ_38_, Aβ_40_ and Aβ_42_ were inversely correlated with chronic WML volume (*p* ≤ 0.01; *p* ≤ 0.05; *p* ≤ 0.05, respectively), but not with acute WML or infarct volumes.

Similarly, van Westen et al. [49] studied the association of cerebral WML with Aβ isoforms and Aβ-PET in 831 subjects with cognitive performance ranging from normal to ADD. The results showed that all Aβ isoforms were consistently inversely correlated with WML, but the CSF Aβ_42/40_ ratio and Aβ-PET were not. These results indicate that the presence of WMLs affects the levels of CSF Aβ_38_, Aβ_40_ and Aβ_42_, but not the CSF Aβ_42/40_ ratio or Aβ-PET.

Finally, Gabelle et al. [14] observed that Aβ_40_ levels were decreased in FTD suggesting that it could represent a diagnostic biomarker in this pathology.

It can therefore be concluded that Aβ_42_, as well as Aβ_38_ and Aβ_40_, might be reduced in CSF due to non-AD related pathologies, but usually, the Aβ_42/40_ ratio, as well as other Aβ ratios, including Aβ_42_, are not affected. These studies therefore point to the usefulness of the CSF Aβ_42/40_ ratio in improving the accuracy of the differential diagnosis in patients with ambiguous biological profiles or with other conditions that might affect the concentrations of the Aβ peptides.

### Effects of pre-analytical handling on Aβ_42_ and the Aβ_42/40_ ratio

Various papers have pointed out pre-analytical and analytical variability between laboratories for the concentration of the Aβ_42_ peptide in CSF [50–54]. Tubes for collection, sample handling and sample storage conditions, in particular, have been noted as critical factors [55, 56]. These factors have also been highlighted as a possible reason for problems linked to inter-centre variability when it comes to analysing CSF biomarkers [57].

The type of sampling and storage tubes used is an important source of variability because of the tendency of Aβ peptides to adsorb on plastic surfaces [21, 54, 56]. It has been proposed that there is parallel adsorption of CSF Aβ_42_ and Aβ_40_ onto the sampling tube surface, regardless of the type of plastic, however with significant differences across different type of plastics [58]. They also suggest that systematic use of the CSF Aβ_42/40_ ratio would provide a complete interpretation of CSF amyloid biomarker results, integrating the impact of plastic tube type.

Lewczuk et al. [58] measured biomarkers of AD (T-tau, P-tau_181_, Aβ_42_, Aβ_40_) in CSF samples in collection tubes made of different materials. The results suggest that the CSF Aβ_42/40_ ratio and CSF P-tau_181_ are much less prone to methodologic error introduced by interactions of the biomarkers’ molecules with the test tube surfaces compared with Aβ_40_ and Aβ_42_ concentrations, similarly to the concentrations of T-tau. The authors concluded that the CSF Aβ_42/40_ ratio is a more reliable biomarker than pure Aβ concentrations, as the Aβ_42/40_ ratio is less altered by interaction with the surface of the collection tubes.

In a similar study by Gervaise-Henry et al. [59], the impact of collection tubes and repetitive freeze/thaw cycles on CSF Aβ_42_ and Aβ_40_ concentrations was investigated. CSF from 35 patients was collected in different polypropylene (PP) tubes and stored at − 80 °C. Samples were also subjected to three successive freeze-thaw cycles. The results showed that CSF Aβ_42_ and Aβ_40_ values were significantly different depending on the type of collection tube and the number of freeze/thaw cycles. Although the calculation of the CSF Aβ_42/40_ ratio eliminated the effect of PP tube-dependent variation, this was not the case for freeze-thaw cycle-associated variation. The authors concluded that the use of Aβ_42/40_ ratio rather than Aβ_42_ alone could contribute toward pre-analytical standardization, thus allowing for the general use of CSF AD biomarkers in routine practice.

Willemse et al. [60] tested several variables as potential confounders influencing adsorption of Aβ peptides on the surfaces of test tubes, including different polypropylene tube brands, volumes, CSF Aβ_42_ concentrations, incubation times, pipettes, vortex intensities and other CSF proteins. They found that every sample transfer from one tube to another resulted in 5% loss of Aβ_42_ concentration, which reached as high as 10% when small volumes were used. This decrease in concentration was, however, similar for Aβ_40_, resulting in stable Aβ_42/40_ ratios over multiple tube transfers. Correspondingly, they conclude that use of the Aβ_42/40_ ratio overcomes the effect of adsorption-derived Aβ concentration loss and can therefore contribute to increased diagnostic accuracy.

Furthermore, Vanderstichele et al. [61] determined that using the CSF Aβ_42/40_ ratio mitigates many of the effects of additional freeze/thaw cycles, tube type and CSF volumes for PP storage tubes. In fact, they concluded that the CSF Aβ_42/40_ ratio is clearly a more robust biomarker than Aβ_42_ toward (pre-) analytical interfering factors. They also observed that the rate of adsorption to PP recipients is higher for Aβ_42_ than for the other amyloid isoforms, such as Aβ_40_, and therefore using the Aβ_42/40_ ratio does not completely eliminate the effects of binding of Aβ to the tube walls of PP tubes. However, they found that ‘low binding recipients’ are able to reduce the binding of Aβ species to the tube walls. They concluded that integration of the Aβ_42/40_ ratio and ‘low-binding tubes’ into guidance criteria may speed up worldwide standardization of CSF biomarker analysis.

In summary, evidence from studies on the effect of pre-analytical handling on biomarkers of AD suggest that use of the CSF Aβ_42/40_ ratio would improve the interpretation of CSF amyloid biomarker results, by reducing the impact of these factors on the final outcome. The use of the CSF Aβ_42/40_ ratio could therefore contribute toward pre-analytical standardization, allowing for the use of CSF AD biomarkers in routine clinical practice.

### Disadvantages of the use of the CSF Aβ_42/40_ ratio

The main disadvantage of the use of the CSF Aβ_42/40_ ratio is economical and not interpretational in nature. Considering the laboratory costs of the AD biomarkers, the inclusion of Aβ_40_ increases the costs by ca. €40–50, which is negligible when considering the total costs of the diagnostic work-up and treatment of patients with suspected AD assessed at specialized memory clinics. Furthermore, the amount of CSF sample needed to perform this additional test is also negligible (5–10 μL, depending on the method). In addition, interpretation of the results when four biomarkers (instead of three) are used is not more complex, when a solid interpretational algorithm is validated and consequently used.

## Conclusion

There is a growing body of evidence that suggests the better diagnostic performance of the CSF Aβ_42/40_ ratio compared to CSF Aβ_42_ alone. In addition, it also appears to be clear that including the CSF Aβ_42/40_ ratio in the clinical workup of MCI patients improves the accuracy of predicting progression to AD.

It has also been shown that both Aβ-PET and CSF biomarkers can identify early amyloid pathology with high accuracy. The CSF Aβ_42/40_ ratio can also better predict abnormal cortical amyloid deposition (visualized with PET) compared with CSF Aβ_42_. This then leads to fewer patients being diagnosed as false positive (low CSF Aβ_42_) or false negative (high CSF Aβ_42_). In addition, there is evidence to suggest that Aβ alterations in CSF occur earlier than they are detectable in Aβ-PET.

Addition of the CSF Aβ_42/40_ ratio to the usual panel of CSF AD biomarkers for patients with ambiguous biological profiles also increases the number of interpretable results.

It has also been found that the use of the CSF Aβ_42/40_ ratio rather than CSF Aβ_42_ alone contributes toward pre-analytical standardization, removing the effects of (pre-)analytical interfering factors, such as tube type, freeze/thaw cycles and CSF volumes.

The working group therefore makes the following recommendations:

1. The Aβ_42/40_ ratio should always be analysed, irrespective of the results of other AD biomarkers, and without paying attention to whether Aβ_42_ is normal or pathologic. This is driven by the fact that the Aβ_42/40_ ratio can equally change the CSF interpretation from ‘normal to pathologic’ as from ‘pathologic to normal’. Analysing the Aβ_42/40_ ratio only in cases with abnormal Aβ_42_ (leaving cases with normal Aβ_42_ without further consideration, i.e. as truly not having amyloidosis) would neglect the former scenario. On the other hand, performing Aβ_42/40_ ratio analysis only in cases with normal Aβ_42_ (with subjects with abnormal Aβ_42_ considered as truly having amyloidosis) would neglect the latter case.
2. It should be mandatory for every laboratory to establish their own specific reference values (cut-offs) for the Aβ_42/40_ ratio and to participate in an internal quality control programme to ensure longitudinal stability in the measurements of Aβ_42_ and Aβ_40_, just as it is mandatory to establish reference values and control quality of all other biomarkers.
3. Following on from (2), it is possible to combine laboratory results obtained on different platforms and from different vendors. The point is only that the reference value for the Aβ_42/40_ ratio must be carefully validated. It is beyond the scope of this paper to make suggestions on statistical methods to calculate reference values and/or their transfer from other laboratories or analytical platforms.

Based on the body of evidence collected here it is the conclusion of the current working group that the CSF Aβ_42/40_ ratio, rather than the absolute value of CSF Aβ_42_, should be used when analysing CSF AD biomarkers to improve the percentage of appropriately diagnosed patients. It is also suggested that the CSF Aβ_42/40_ ratio could therefore be used as a proxy for amyloid status in AD clinical trials and eventually in clinical care settings.

## Abbreviations

AD: Alzheimer’s Disease
ADD: AD dementia
AUC: Area under the ROC curve
Aβ_42_: Amyloid β 42
Aβ_42/40_ Ratio: Concentration ratio of Aβ_42_ to Aβ_40_
Aβ-PET: Aβ positron emission tomography
CSF: Cerebrospinal Fluid
DLB: Dementia with Lewy Bodies
FTD: Frontotemporal Dementia
MCI: Mild cognitive impairment
MCI-AD: MCI evolving into ADD
MCI-MCI: Stable MCI
non-AD: Other types of dementia
PDD: Parkinson’s Disease Dementia
PiB: Pittsburgh compound B
PLM_R_-scale: PLM ratio scale
PLM-scale: Paris-Lille-Montpellier scale
P-tau_181_: Tau phosphorylated at threonine 181
ROC: Receiver Operating Characteristic
SCD: Subjective cognitive decline
T-tau: Total Tau
VaD: Subcortical vascular dementia
WML: White matter lesions

## References

[1] Sperling RA, Aisen PS, Beckett LA (2011). Toward defining the preclinical stages of Alzheimer’s disease: recommendations from the National Institute on Aging-Alzheimer’s Association workgroups on diagnostic guidelines for Alzheimer’s disease. Alzheimers Dement.

[2] Albert MS, DeKosky ST, Dickson D (2011). The diagnosis of mild cognitive impairment due to Alzheimer’s disease: recommendations from the National Institute on Aging-Alzheimer’s Association workgroups on diagnostic guidelines for Alzheimer’s disease. Alzheimers Dement.

[3] McKhann GM, Knopman DS, Chertkow H (2011). The diagnosis of dementia due to Alzheimer’s disease: recommendations from the National Institute on Aging and the Alzheimer’s Association workgroup. Alzheimers Dement.

[4] Peskind ER, Riekse R, Quinn JF (2005). Safety and acceptability of the research lumbar puncture. Alzheimer Dis Assoc Disord.

[5] Lewczuk Piotr, Riederer Peter, O’Bryant Sid E., Verbeek Marcel M., Dubois Bruno, Visser Pieter Jelle, Jellinger Kurt A., Engelborghs Sebastiaan, Ramirez Alfredo, Parnetti Lucilla, Jack Clifford R., Teunissen Charlotte E., Hampel Harald, Lleó Alberto, Jessen Frank, Glodzik Lidia, de Leon Mony J., Fagan Anne M., Molinuevo José Luis, Jansen Willemijn J., Winblad Bengt, Shaw Leslie M., Andreasson Ulf, Otto Markus, Mollenhauer Brit, Wiltfang Jens, Turner Martin R., Zerr Inga, Handels Ron, Thompson Alexander G., Johansson Gunilla, Ermann Natalia, Trojanowski John Q., Karaca Ilker, Wagner Holger, Oeckl Patrick, van Waalwijk van Doorn Linda, Bjerke Maria, Kapogiannis Dimitrios, Kuiperij H. Bea, Farotti Lucia, Li Yi, Gordon Brian A., Epelbaum Stéphane, Vos Stephanie J. B., Klijn Catharina J. M., Van Nostrand William E., Minguillon Carolina, Schmitz Matthias, Gallo Carla, Lopez Mato Andrea, Thibaut Florence, Lista Simone, Alcolea Daniel, Zetterberg Henrik, Blennow Kaj, Kornhuber Johannes (2017). Cerebrospinal fluid and blood biomarkers for neurodegenerative dementias: An update of the Consensus of the Task Force on Biological Markers in Psychiatry of the World Federation of Societies of Biological Psychiatry. The World Journal of Biological Psychiatry.

[6] Fagan AM, Perrin RJ (2012). Upcoming candidate cerebrospinal fluid biomarkers of Alzheimer’s disease. Biomark Med.

[7] Lewczuk P, Kornhuber J (2011). Neurochemical dementia diagnostics in Alzheimer’s disease: where are we now and where are we going?. Expert Rev Proteomics.

[8] Hampel H, Burger K, Teipel SJ (2008). Core candidate neurochemical and imaging biomarkers of Alzheimer’s disease. Alzheimers Dement.

[9] Hansson O, Zetterberg H, Buchhave P (2006). Association between CSF biomarkers and incipient Alzheimer’s disease in patients with mild cognitive impairment: a follow-up study. Lancet Neurol.

[10] Mattsson N, Zetterberg H, Hansson O (2009). CSF biomarkers and incipient Alzheimer disease in patients with mild cognitive impairment. JAMA.

[11] Petersen RC (2004). Mild cognitive impairment as a diagnostic entity. J Intern Med.

[12] Shoji M, Matsubara E, Kanai M, Watanabe M, Nakamura T, Tomidokoro Y (1998). Combination assay of CSF tau, A beta 1-40 and A beta 1-42(43) as a biochemical marker of Alzheimer’s disease. J Neurol Sci.

[13] Lewczuk P, Esselmann H, Otto M (2004). Neurochemical diagnosis of Alzheimer’s dementia by CSF Abeta42, Abeta42/Abeta40 ratio and total tau. Neurobiol Aging.

[14] A G, Roche S, Gény C (2011). Decreased sAβPPβ, Aβ38, and Aβ40 cerebrospinal fluid levels in frontotemporal dementia. J Alzheimers Dis.

[15] Wiltfang J, Esselmann H, Bibl M, Hüll M, Hampel H, Kessler H (2007). Amyloid beta peptide ratio 42/40 but not A beta 42 correlates with phospho-Tau in patients with low- and high-CSF A beta 40 load. J Neurochem.

[16] Slaets S, Le Bastard N, Martin JJ, Sleegers K, Van Broeckhoven C, De Deyn PP (2013). Cerebrospinal fluid Aβ1-40 improves differential dementia diagnosis in patients with intermediate P-tau_181P_ levels. J Alzheimers Dis.

[17] Nutu M, Zetterberg H, Londos E, Minthon L, Nägga K, Blennow K (2013). Evaluation of the cerebrospinal fluid amyloid-β1-42/amyloid-β1-40 ratio measured by alpha-LISA to distinguish Alzheimer's disease from other dementia disorders. Dement Geriatr Cogn Disord.

[18] Baldeiras I, Santana I, Leitão MJ (2015). Cerebrospinal fluid Aβ40 is similarly reduced in patients with frontotemporal lobar degeneration and Alzheimer’s disease. J Neurol Sci.

[19] Dumurgier J, Schraen S, Gabelle A (2015). Cerebrospinal fluid amyloid-β 42/40 ratio in clinical setting of memory centers: a multicentric study. Alzheimers Res Ther.

[20] Sauvée M, DidierLaurent G, Latarche C, Escanyé MC, Olivier JL, Malaplate-Armand C (2014). Additional use of Aβ_42_/Aβ_40_ ratio with cerebrospinal fluid biomarkers P-tau and Aβ_42_ increases the level of evidence of Alzheimer’s disease pathophysiological process in routine practice. J Alzheimers Dis.

[21] Lewczuk P, Lelental N, Spitzer P, Maler JM, Kornhuber J (2015). Amyloid-β 42/40 cerebrospinal fluid concentration ratio in the diagnostics of Alzheimer’s disease: validation of two novel assays. J Alzheimers Dis.

[22] Spies PE, Slats D, Sjögren JM, Kremer BP, Verhey FR, Rikkert MG (2010). The cerebrospinal fluid amyloid beta42/40 ratio in the differentiation of Alzheimer’s disease from non-Alzheimer’s dementia. Curr Alzheimer Res.

[23] Struyfs H, Van Broeck B, Timmers M, Fransen E, Sleegers K, Van Broeckhoven C (2015). Diagnostic accuracy of cerebrospinal fluid amyloid-β isoforms for early and differential dementia diagnosis. J Alzheimers Dis.

[24] Janelidze S, Zetterberg H, Mattsson N, Palmqvist S, Vanderstichele H, Lindberg O (2016). CSF Aβ42/Aβ40 and Aβ42/Aβ38 ratios: better diagnostic markers of Alzheimer disease. Ann Clin Transl Neurol.

[25] Bousiges O, Cretin B, Lavaux T, Philippi N, Jung B, Hezard S (2016). Diagnostic value of cerebrospinal fluid biomarkers (Phospho-Tau181, total-Tau, Aβ42, and Aβ40) in prodromal stage of Alzheimer’s disease and dementia with Lewy bodies. J Alzheimers Dis.

[26] Lehmann S, Delaby C, Boursier G (2018). Relevance of Aβ42/40 ratio for detection of Alzheimer disease pathology in clinical routine: the PLMR scale. Front Aging Neurosci.

[27] Baldeiras I, Santana I, Leitão MJ (2018). Addition of the Aβ42/40 ratio to the cerebrospinal fluid biomarker profile increases the predictive value for underlying Alzheimer’s disease dementia in mild cognitive impairment. Alz Res Ther.

[28] Dorey A, Perret-Liaudet A, Tholance Y, Fourier A, Quadrio I (2015). Cerebrospinal fluid Aβ40 improves the interpretation of Aβ42 concentration for diagnosing Alzheimer’s disease. Front Neurol.

[29] Lewczuk P, Zimmermann R, Wiltfang J, Kornhuber J (2009). Neurochemical dementia diagnostics: a simple algorithm for interpretation of the CSF biomarkers. Neural Transm (Vienna).

[30] Lewczuk P, Kornhuber J, Toledo JB, Trojanowski JQ, Knapik-Czajka M, German Dementia Competence Network (2015). Validation of the Erlangen score algorithm for the prediction of the development of dementia due to Alzheimer’s disease in pre-dementia subjects. J Alzheimers Dis.

[31] Hansson O, Zetterberg H, Buchhave P, Andreasson U, Londos E, Minthon L, Blennow K (2007). Prediction of Alzheimer’s disease using the CSF Abeta42/Abeta40 ratio in patients with mild cognitive impairment. Dement Geriatr Cogn Disord.

[32] Hertze J, Minthon L, Zetterberg H, Vanmechelen E, Blennow K, Hansson O (2010). Evaluation of CSF biomarkers as predictors of Alzheimer’s disease: a clinical follow-up study of 4.7 years. J Alzheimers Dis.

[33] Parnetti L, Chiasserini D, Eusebi P, Giannandrea D, Bellomo G, De Carlo C (2012). Performance of Aβ1-40, Aβ1-42, total tau, and phosphorylated tau as predictors of dementia in a cohort of patients with mild cognitive impairment. J Alzheimers Dis.

[34] Frölich L, Peters O, Lewczuk P (2017). Incremental value of biomarker combinations to predict progression of mild cognitive impairment to Alzheimer’s dementia. Alzheimers Res Ther.

[35] Seppala TT, Nerg O, Koivisto AM (2012). CSF biomarkers for Alzheimer disease correlate with cortical brain biopsy findings. Neurology.

[36] Wolk DA, Grachev ID, Buckley C (2011). Association between in vivo fluorine 18-labeled flutemetamol amyloid positron emission tomography imaging and in vivo cerebral cortical histopathology. Arch Neurol.

[37] Lewczuk P, Kornhuber J (2016). Do we still need positron emission tomography for early Alzheimer’s disease diagnosis?. Brain.

[38] Blennow K, Hampel H, Weiner M, Zetterberg H (2010). Cerebrospinal fluid and plasma biomarkers in Alzheimer disease. Nat Rev Neurol.

[39] Buerger K, Ewers M, Pirttila T (2006). CSF phosphorylated tau protein correlates with neocortical neurofibrillary pathology in Alzheimer’s disease. Brain.

[40] Blennow K, Wallin A, Hager O (1993). Low frequency of post-lumbar puncture headache in demented patients. Acta Neurol Scand.

[41] Lewczuk P, Matzen A, Blennow K, Parnetti L, Molinuevo JL, Eusebi P (2017). Cerebrospinal fluid Aβ42/40 corresponds better than Aβ42 to amyloid PET in Alzheimer’s disease. J Alzheimers Dis.

[42] Mattsson N, Insel PS, Donohue M, Landau S, Jagust WJ, Shaw LM (2015). Independent information from cerebrospinal fluid amyloid-beta and florbetapir imaging in Alzheimer’s disease. Brain.

[43] Palmqvist S, Mattsson N, Hansson O, Alzheimer’s Disease Neuroimaging Initiative (2016). Cerebrospinal fluid analysis detects cerebral amyloid-beta accumulation earlier than positron emission tomography. Brain.

[44] Janelidze S, Pannee J, Mikulskis A (2017). Concordance between different amyloid immunoassays and visual amyloid positron emission tomographic assessment. JAMA Neurol.

[45] Leuzy A, Chiotis K, Hasselbalch SG (2016). Pittsburgh compound B imaging and cerebrospinal fluid amyloid-β in a multicentre European memory clinic study. Brain.

[46] Schindler Suzanne E., Gray Julia D., Gordon Brian A., Xiong Chengjie, Batrla-Utermann Richard, Quan Marian, Wahl Simone, Benzinger Tammie L.S., Holtzman David M., Morris John C., Fagan Anne M. (2018). Cerebrospinal fluid biomarkers measured by Elecsys assays compared to amyloid imaging. Alzheimer's & Dementia.

[47] Thal DR, Attems J, Ewers M (2014). Spreading of amyloid, tau, and microvascular pathology in Alzheimer’s disease: findings from neuropathological and neuroimaging studies. J Alzheimers Dis.

[48] Selnes P, Blennow K, Zetterberg H (2010). Effects of cerebrovascular disease on amyloid precursor protein metabolites in cerebrospinal fluid. Cerebrospinal Fluid Res.

[49] van Westen D, Lindqvist D, Blennow K (2016). Cerebral white matter lesions – associations with Aβ isoforms and amyloid PET. Sci Rep.

[50] Verwey NA, van der Flier WM, Blennow K, Clark C, Sokolow S, De Deyn PP (2009). A worldwide multicentre comparison of assays for cerebrospinal fluid biomarkers in Alzheimer’s disease. Ann Clin Biochem.

[51] Perret-Liaudet A, Pelpel M, Tholance Y, Dumont B, Vanderstichele H, Zorzi W (2012). Risk of Alzheimer’s disease biological misdiagnosis linked to cerebrospinal collection tubes. J Alzheimers Dis.

[52] Leitão MJ, Baldeiras I, Herukka S-K, Pikkarainen M, Leinonen V, Simonsen AH (2015). Chasing the effects of pre-analytical confounders – a multicenter study on CSF-AD biomarkers. Front Neurol.

[53] Le Bastard N, De Deyn PP, Engelborghs S (2015). Importance and impact of preanalytical variables on Alzheimer disease biomarker concentrations in cerebrospinal fluid. Clin Chem.

[54] Fourier A, Portelius E, Zetterberg H, Blennow K, Quadrio I, Perret-Liaudet A (2015). Pre-analytical and analytical factors influencing Alzheimer’s disease cerebrospinal fluid biomarker variability. Clin Chim Acta.

[55] Bjerke Maria, Portelius Erik, Minthon Lennart, Wallin Anders, Anckarsäter Henrik, Anckarsäter Rolf, Andreasen Niels, Zetterberg Henrik, Andreasson Ulf, Blennow Kaj (2010). Confounding Factors Influencing Amyloid Beta Concentration in Cerebrospinal Fluid. International Journal of Alzheimer's Disease.

[56] Del Campo M, Mollenhauer B, Bertolotto A, Engelborghs S, Hampel H, Simonsen AH (2012). Recommendations to standardize preanalytical confounding factors in Alzheimer’s and Parkinson’s disease cerebrospinal fluid biomarkers: an update. Biomark Med.

[57] Mattsson N, Andreasson U, Persson S, Carrillo MC, Collins S, Chalbot S (2013). CSF biomarker variability in the Alzheimer’s Association QC program work group. Alzheimers Dement.

[58] Lewczuk P, Beck G, Esselmann H, Bruckmoser R, Zimmermann R, Fiszer M (2006). Effect of sample collection tubes on cerebrospinal fluid concentrations of tau proteins and amyloid beta peptides. Clin Chem.

[59] Gervaise-Henry C, Watfa G, Albuisson E, Kolodziej A, Dousset B, Olivier JL (2017). Cerebrospinal fluid Aβ_42_/Aβ_40_ as a means to limiting tube- and storage-dependent pre-analytical variability in clinical setting. J Alzheimers Dis.

[60] Willemse E, van Uffelen K, Brix B, Engelborghs S, Vanderstichele H, Teunissen C (2017). How to handle adsorption of cerebrospinal fluid amyloid β (1-42) in laboratory practice? Identifying problematic handlings and resolving the issue by use of the Aβ42/Aβ40 ratio. Alzheimers Dement.

[61] Vanderstichele HM, Janelidze S, Demeyer L (2016). Optimized standard operating procedures for the analysis of cerebrospinal fluid Aβ42 and the ratios of Aβ isoforms using low protein binding tubes. J Alzheimers Dis.

